# Causes of Death in Chinese Patients with Multiple System Atrophy

**DOI:** 10.14336/AD.2017.0711

**Published:** 2018-02-01

**Authors:** LingYu Zhang, Bei Cao, Yutong Zou, Qian-Qian Wei, RuWei Ou, Wanglin Liu, Bi Zhao, Jing Yang, Ying Wu, HuiFang Shang

**Affiliations:** ^1^Department of Neurology, West China Hospital, Sichuan University, Chengdu, Sichuan, China; ^2^West China School of Medicine, Sichuan University, 610041, Chengdu, Sichuan, China

**Keywords:** multiple system atrophy, cause of death, nocturnal stridor

## Abstract

The objective of this study was to explore the causes of death in Chinese patients with multiple system atrophy (MSA) as well as differences in the cause of death according to sex, subtype, disease onset, and whether the disease was accompanied by nocturnal stridor. A total of 131 MSA patients were enrolled and followed up once every year until their deaths. Clinical information was collected by neurologists, and the cause of death of the MSA patients was obtained from the patients’ relatives or caregivers. The current study included 62 MSA with predominant parkinsonism (MSA-P) and 69 MSA with predominant cerebellar ataxia (MSA-C) patients. Median survival time from disease onset to death of the MSA patients was 5.59 years. The most common cause of death was respiratory infection (65.6%). The second most common cause of death was sudden death (14.5%). Other causes included nutritional disorder due to dysphagia (9.2%), urinary tract infection (3.1%), suicide (2.3%), choking (1.5%), cerebrovascular accident (1.5%), myocardial infarction (1.5%), and lymphoma (0.8%). We found that sudden death was more likely to occur in patients with nocturnal stridor than in those without (P<0.001). There were no significant differences in the cause of death according to subtype, sex, or onset symptoms (autonomic failure or motor symptoms). Sudden death is a relatively common cause of death in MSA patients, second only to respiratory infection, especially in patients with nocturnal stridor. The information provided by our study may help to provide better medical care to MSA patients.

Multiple system atrophy (MSA) is a sporadic, adult-onset neurodegenerative disease with a heterogeneous combination of autonomic failure, cerebellar ataxia, parkinsonian features, and pyramidal signs [1]. The estimated annual incidence of MSA is about 0.6 per 100 000 per year, reaching 3 per 100 000 per year in the Caucasian population older than 50 years [1]. The crude prevalence rate was 13 per 100 000 for MSA in a rural Japanese district after aging adjustment [2]. There are no epidemiological data of MSA patients in China at present. MSA-P is common in Western countries, while MSA-C is more common in Asian countries [1]. Patients with MSA have a shorter survival time, with a median survival time (from symptom onset to death) of approximately 6~10 years [3-9]. Varied factors have been presented to predict the survival of patients with MSA, such as autonomic failure, the parkinsonian variant of MSA, and older age of onset [8-10]. Patients with MSA have been reported to die of respiratory infection, sudden death, choking, cancer, suicide, and stroke, among other causes [5, 11-18]. The frequency of different causes of death in MSA is inconsistent across studies. Some studies found that the leading cause of death was sudden death (26% ~ 70%) in MSA [11, 13, 14, 16]; others reported that respiratory infection was the most common cause of death in MSA [12, 18]. Only two small studies focused on the relationship between stridor and death, suggesting that stridor increased the risk of death [12, 13]. A few patients with MSA have been reported to die of suicide.

However, the sample sizes of these studies that focused on the causes of death in MSA were relatively small. In addition, some studies used the previous diagnostic criteria and classification of MSA, but there is a new consensus on MSA [19]. Whether the causes of death in MSA patients differ according to sex, subtypes, and onset symptoms remains largely unknown. Whether the causes of death differ between patients with and without nocturnal stridor is also unknown. Better understanding of the causes of death in patients with MSA could help to provide better medical advice and care to the MSA patients at the end of their lives. Thus, exploring the causes of death in MSA patients is a matter of great significance.

## MATERIALS AND METHODS

### Patients and clinical data

One hundred and thirty-one patients with MSA were recruited from the Department of Neurology, West China Hospital of Sichuan University. These patients were followed up every year by face-to-face interview or by telephone interview (if they could not come to the hospital) until their deaths between 2010 and 2016. All MSA patients met the second consensus criteria for probable MSA [20]. All patients received brain MRI scans and other supplemental tests, such as spinal cerebellar ataxia genetic tests (SCA1, 2, 3, 6, 7), immunological tests, and tumor marker tests to exclude other neurological diseases. All patients were diagnosed in the department by movement specialists (HuiFang Shang, Ying Wu, and Bi Zhao). Neurologists (LingYu Zhang, Bei Cao, QianQian Wei, RuWei Ou, and Jing Yang who passed the consistency check before conducted the study) collected the clinical information of the MSA patients, including age, sex, age of onset, and onset symptoms and so on during face-to face interviews when they first came to our department; these data were input into our MSA patient database. The clinical and demographic information of the MSA patients during follow-up were also recorded in our database. Disease onset was defined as the initial presentation of any motor problems, whether parkinsonian or cerebellar, or autonomic features, with the exception of male erectile dysfunction [20]. Survival time was defined as the time of the disease onset until death. Whether the patients had nocturnal stridor was determined by asking the patients and a family member (bed partner). The causes of deaths of the MSA patients were obtained from the patients’ relatives or caregivers according to the death certification or the latest hospital record.

The present study was approved by the ethics committee of the West China Hospital of Sichuan University. All subjects signed informed consent forms.

### Statistical analysis

All continuous data, including the age of onset, disease duration, and age of death, were presented as the mean ± standard deviation. Differences between subgroups were evaluated using the chi-square test or Fisher exact test. Kaplan-Meyer analysis was used to establish the survival curve of our MSA patients. All the data analyses were performed using SPSS 22.0 (IBM, Chicago, IL). A value of P < 0.05 was considered statistically significant.

**Table 1:** Demographic and clinical characteristics of the patients with MSA.

Variables	MSA
Diagnosis (MSA-P/MSA-C)	62/69(47.3%/52.7%)
Sex (male/female)	73/58(55.7%/44.3%)
Age of onset	56.94 ± 7.50
Disease duration	5.61 ± 1.77
Age of death	62.55 ± 7.50

MSA, multiple system atrophy; MSA-P, multiple system atrophy with predominately parkinsonism; MSA-C, multiple system atrophy with predominately cerebellar ataxia.

## RESULTS

**Figure 1.**
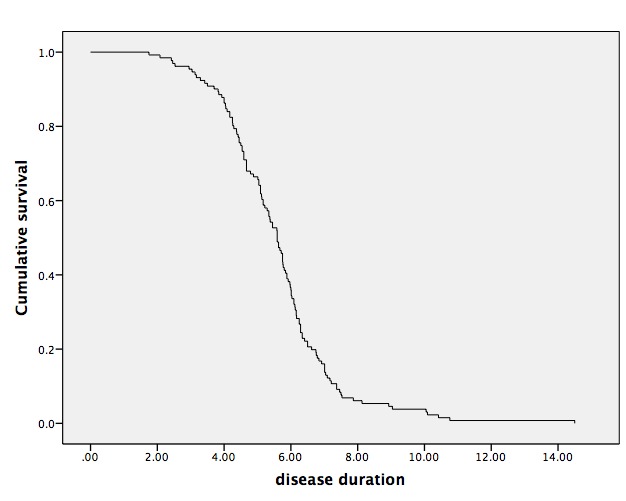
Kaplan-Meier survival curve of the 131 MSA patients Median survival from symptom onset to death was 5.59 years.

The demographic and clinical characteristics of the patients with MSA are presented in Table 1. Among the 131 MSA patients, including 73 male and 58 female patients, 62 were MSA-P and 69 were MSA-C patients. The mean age of onset was 56.94 ± 7.50 years, the mean survival time was 5.61 ± 1.77 years, and the mean age of death was 62.55 ± 7.50 years. The median survival from disease onset to death was 5.59 years (Fig. 1).

As shown in Table 2, the most common cause of death was respiratory infection (86,65.6%), which included aspiration pneumonia. The second most common cause of death was sudden death (19, 14.5%), which occurred in 12 patients (9.2%) during the day and in 7 patients (5.3%) at night. Twelve patients (9.2%) died of a nutritional disorder due to dysphagia, 4 patients (3.1%) died of a urinary tract infection, 3 patients (2.3%) died of suicide, 2 patients (1.5%) died of choking, 2 patients (1.5%) died of cerebrovascular accident, 2 patients (1.5%) died of myocardial infarction, and one patient (0.8%) died of lymphoma.

**Table 2:** Cause of death of MSA patients according to gender and subtype.

Cause of death	Total (131) N (%)	Female (58) N (%)	Male (73) N (%)	P-value#	MSA-C (69) N (%)	MSA-P (62) N (%)	P-value#
Respiratory infection	86(65.6)	38(65.5)	48(65.8)	0.977	41(59.4)	45(72.6)	0.113
Choking	2(1.5)	1(1.7)	1(1.4)	1.000	1(1.4)	1(1.6)	1.000
Urinary tract infection	4(3.1)	2(3.4)	2(2.7)	1.000	3(4.3)	1(1.6)	0.689
Nutritional disorder	12(9.2)	8(13.8)	4(5.5)	0.101	8(11.6)	4(6.5)	0.308
Lymphoma	1(0.8)	0(0.0)	1(1.4)	1.000	1(1.4)	0(0.0)	1.000
Cerebrovascular accident	2(1.5)	0(0.0)	2(2.7)	0.503	1(1.4)	1(1.6)	1.000
Myocardial infarction	2(1.5)	0(0.0)	2(2.7)	0.503	2(2.9)	0(0.0)	0.498
Suicide	3(2.3)	0(0.0)	3(4.1)	0.330	3(4.3)	0(0.0)	0.282
Sudden death	19(14.5)	9(15.5)	10(13.7)	0.769	9(13.0)	10(16.1)	0.617
Nighttime	7(5.3)	1(1.7)	6(8.2)	0.211	5(7.2)	2(3.2)	0.527
Daytime	12(9.2)	8(13.8)	4(5.5)	0.101	4(5.8)	8(12.9)	0.159

# Chi-square or Fischer exact test.1

MSA, multiple system atrophy; MSA-P, multiple system atrophy with predominately parkinsonism; MSA-C, multiple system atrophy with predominately cerebellar ataxia.

**Table 3:** Cause of death of MSA patients according to disease onset.

Cause of death	Total (131) N (%)	Autonomic failure (81) N (%)	Cerebellar ataxia or parkinsonism (50) N (%)	P-value#
Respiratory infection	86(65.6)	50(61.7)	36(72.0)	0.229
Choking	2(1.5)	1(1.2)	1(2.0)	1.000
Urinary tract infection	4(3.1)	4(4.9)	0(0.0)	0.283
Nutritional disorder	12(9.2)	9(11.1)	3(6.0)	0.501
Lymphoma	1(0.8)	0(0.0)	1(2.0)	0.382
Cerebrovascular accident	2(1.5)	2(2.5)	0(0.0)	0.524
Myocardial infarction	2(1.5)	0(0.0)	2(4.0)	0.144
Suicide	3(2.3)	3(3.7)	0(0.0)	0.438
Sudden death	19(14.5)	12(14.8)	7(14.0)	0.898
Nighttime	7(5.3)	4(4.9)	3(6.0)	1.000
Daytime	12(9.2)	8(9.9)	4(8.0)	0.960

# Chi-square or Fischer exact test.

MSA, multiple system atrophy.

There were no significant differences in the causes of death between the MSA-P and MSA-C patients (Table 2), between the male and female patients (Table 2), or between the patients with autonomic failure or motor symptoms (parkinsonism or cerebellar ataxia symptoms) as onset symptoms (Table 3). Sudden death was more likely to occur in patients with nocturnal stridor than in those without (42.9% vs. 9.1%, P < 0.001), regardless of whether death occurred during the day or night. The percentage of respiratory infection was higher in patients without nocturnal stridor than in patients with nocturnal stridor (70.9% vs. 38.1%, P = 0.004). The remaining causes of death did not significantly different between the patients with and without nocturnal stridor.

**Table 4:** Cause of death of MSA patient according to whether the disease was accompanied by nocturnal stridor.

Cause of death	Total (131) N (%)	Nocturnal stridor		P-value#
		No (110) N (%)	Yes (21) N (%)	
Respiratory infection	86(65.6)	78(70.9)	8(38.1)	0.004*
Choking	2(1.5)	2(1.8)	0(0.0)	1.000
Urinary tract infection	4(3.1)	4(3.6)	0(0.0)	1.000
Nutritional disorder	12(9.2)	8(7.3)	4(19.0)	0.193
Lymphoma	1(0.8)	1(0.9)	0(0.0)	1.000
Cerebrovascular accident	2(1.5)	2(1.8)	0(0.0)	1.000
Myocardial infarction	2(1.5)	2(1.8)	0(0.0)	1.000
Suicide	3(2.3)	3(2.7)	0(0.0)	1.000
Sudden death	19(14.5)	10(9.1)	9(42.9)	0.000*
Nighttime	7(5.3)	3(2.7)	4(19.0)	0.012*
Daytime	12(9.2)	7(6.4)	5(23.8)	0.033*

# Chi-square or Fischer exact test.

* Significant difference.

MSA, multiple system atrophy

## DISCUSSION

This is a prospective study in that we followed up 131 MSA patients until their deaths. Our present study showed that the leading cause of death in the MSA patients was respiratory infection, followed by sudden death and nutritional disorders. Sudden death was more common in the patients with nocturnal stridor than in those without. The median survival time from symptom onset to death was 5.59 years, which is in line with an Icelandic study that found a median survival time of 5.7 years [6] and a meta-analysis of 433 patients with autopsy-proven MSA that reported a survival time of 6.2 years [3]. However, it was shorter than the findings of other studies that reported a median survival time ranging from 7 to 10 years [4, 5, 7-9]. Several reasons may explain such a discrepancy. It was proved that the early development of autonomic dysfunction is a predictive factor for shorter survival in patients with MSA [5, 21]. The percentage of MSA patients who had autonomic failure (61.8%) as the initial symptom in the current study was higher than that of other studies (23%~50%) [4, 5, 7, 8]. In addition, our patients mainly came from southwest China, where the economy is underdeveloped.

The leading cause of death was respiratory infection (86, 65.6%), which is in accordance with previous studies [12, 18]. Flabeau, et al. found that aspiration pneumonia (9/15, 60%) was the most common cause of death during follow-up, as it was the cause of death of 15 of the 28 enrolled MSA patients [18]. In the late stage of the disease, almost all patients were bedridden at home and their autoimmune function was decreased, which increases the risk of infection. Of the 86 MSA patients who died of respiratory infection, some patients had a history of swallowing aspiration, which increases the risk of respiratory infection.

The second most common cause of death was sudden death (14.5%). In agreement with another study [13], we found that patients with nocturnal stridor were more likely to die of sudden death than those without. Laryngeal dysfunction probably plays an important role in the sudden death of stridor patients [13]. Patients with nocturnal stridor experience an increased risk of developing obstructive sleep apnea (OSA) [22]. Although most of our patients in the current study did not receive the polysomnography (PSG) test, our previous study of MSA patients with PSG investigation found that 70% of MSA patients have OSA [23]. OSA increases the risk of nighttime death in MSA patients [12]. We should advise MSA patients with nocturnal stridor to identify OSA using PSG, and such patients should use a non-invasive ventilator (NIV) during sleep to prevent sleep apnea. Tracheostomy can be proposed to patients with MSA who have stridor to reduce the risk of sudden death [12]. Upper airway dysfunction associated with autonomic failure and dysfunction of the medullary serotonergic system, which regulates the cardiovascular and respiratory systems, could also be responsible for sudden death in patients with MSA [24, 25] since sudden death also occurred during the daytime.

In addition, 9.2% of the MSA patients died of a nutritional disorder. The progression of cerebellar dysfunction and overlapping parkinsonism will worsen tongue movements, and in the late stage of the disease, swallowing function of the oral phase (bolus transport and bolus holding) is remarkably disturbed [26]. Several patients died of a nutritional disorder, with severe weight loss at the late stage of the disease due to dysphagia since they were unable to tolerate the discomfort of a nasogastric tube and did not accept percutaneous endoscopic gastrostomy (PEG) treatment. In addition, choking (1.5%) was one of the causes of death in our patients. Caregivers should pay attention to patients’ dietary traits to reduce the risk of choking. Nasogastric tube or PEG should be recommended to patients when it is necessary. Beyond that, parenteral nutrition can also be taken into consideration.

Overall, 3.1% of the patients died of a urinary tract infection. Neurogenic urinary dysfunction, including urinary incontinence, unexplained urinary urgency or frequency or incomplete bladder emptying, is one of the diagnostic criteria for MSA [20]. Many patients complained of urinary dysfunction, and some suffered a lower urinary tract infection. However, although clean intermittent self-catheterization (CISC) is the recommended treatment for most MSA patients with urinary retention, only a few patients took our advice to perform CISC at home, by either themselves or their family member. Patients who have difficulty in performing CISC can also take urethra-oriented medication and surgery into account [27].

Although the current study found that only 3 patients died of suicide, our previous study showed that not only disease severity but also severe non-motor symptoms (especially mood disorder) had a negative impact on the quality of life in MSA [28]. Furthermore, depression has been reported to be common in MSA [29, 30]. We should not only keep an eye on the motor symptoms but also pay attention to mood disorders to avoid the occurrence of suicide.

Furthermore, our present study showed that sex, subtype, and onset symptoms did not influence the cause of death in patients with MSA. However, this study also had limitations. The patients were registered from a single center and may not represent the Chinese MSA patients as a whole, so it needs further multicenter validation in China. Most of our patients did not receive the PSG test, so some patients who had nocturnal stridor could have been missed.

## Conclusion

In conclusion, this is the first large study to evaluate the clinical causes of death in Chinese patients with MSA. Although there were several causes of death in MSA, respiratory infection was the major cause. Sudden death was the second most common cause of mortality and was especially common in patients with nocturnal stridor.
